# Simultaneous Determination of Drugs Affecting Central Nervous System (CNS) in Bulk and Pharmaceutical Formulations Using Multivariate Curve Resolution-Alternating Least Squares (MCR-ALS)

**DOI:** 10.1155/2020/1684172

**Published:** 2020-02-11

**Authors:** Heba Shaaban, Ahmed Mostafa, Bushra Al-Zahrani, Bushra Al-Jasser, Raghad Al-Ghamdi

**Affiliations:** Department of Pharmaceutical Chemistry, College of Clinical Pharmacy, Imam Abdulrahman Bin Faisal University, King Faisal Road, P.O. Box 1982, Dammam 31441, Saudi Arabia

## Abstract

The quality of medications is important to maintain the overall health care of patients. This study aims to develop and validate a spectrophotometric method using multivariate curve resolution-alternating least squares (MCR-ALS) with correlation constraint for simultaneous resolution and quantification of selected drugs affecting the central nervous system (imipramine, carbamazepine, chlorpromazine, haloperidol, and phenytoin) in different pharmaceutical dosage forms. Figures of merit such as root-mean-square error of prediction, bias, standard error of prediction, and relative error of prediction for the developed method were calculated. High values of correlation coefficients ranged between 0.9993 and 0.9998 reflected high predictive ability of the developed method. The results are linear in the concentration range of 0.3–5 *μ*g/mL for carbamazepine, 0.3–15 *μ*g/mL for chlorpromazine, 0.5–10 *μ*g/mL for haloperidol, 0.5–10 *μ*g/mL for imipramine, and 3–20 *μ*g/mL for phenytoin. The optimized method was successfully applied for the analysis of the studied drugs in their pharmaceutical products without any separation step. The optimized method was also compared with a reported HPLC method using Student's *t* test and *F* ratio at 95% confidence level, and the results showed no significant difference regarding accuracy and precision. The proposed chemometric method is fast, reliable, and cost-effective and can be used as an eco-friendly alternative to chromatographic techniques for the analysis of the studied drugs in commercial pharmaceutical products.

## 1. Introduction

Central nervous system (CNS) disorders are a growing medical concern worldwide. Many people suffer from CNS disorders, and this number is increasing. Thus, the use of drugs affecting CNS has been increased as well. The studied pharmaceuticals are among the most commonly used CNS drugs, and therefore developing fast, simple, and eco-friendly methods for their simultaneous determination is needed.

Chlorpromazine (CHZ) is used in suppressing excitement, agitation, and other psychomotor disorders and can be also used as antiemetic and in the treatment of intractable hiccup. [1]. Carbamazepine (CRZ) is indicated to treat partial seizures, tonic-clonic seizures, trigeminal neuralgia pain, and psychiatric disorders such as manic-depressive illness [2]. Phenytoin (PTN) is used for the control of certain types of seizures and prevention of seizures happening after neurosurgeries [3]. Haloperidol (HAL) is indicated for the treatment of schizophrenia. It is also the drug of choice of Tourette syndrome [4]. Imipramine (IMP) inhibits serotonin and norepinephrine reuptake [5].

Several methods have been developed for the analysis of these drugs, either individually or in combination. For example, partial least squares (PLS) and MCR-ALS were used for the simultaneous determination of carbamazepine along with diclofenac, naproxen, and other anti-inflammatory drugs [6]. HPLC-DAD was used for the analysis of carbamazepine and phenobarbital in human serum samples and was further evaluated by the MCR-ALS method [7]. MCR was also used to obtain information about the polymorphic transformation of carbamazepine tablets during the heating process [8]. HPLC was employed for the determination of HAL, its three metabolites, and two butyrophenone-type neuroleptics in phosphate-buffered saline. [9]. PTN, CRZ, primidone, phenobarbital, and two active metabolites were determined simultaneously using HPLC [10]. HPLC was also employed for the analysis of the studied analytes [11].

Greening analytical procedures is of paramount importance in order to minimize the negative environmental impacts [12, 13]. Green analytical chemistry aims at substituting nongreen analytical methods with more eco-friendly alternatives that consume and generate less toxic solvents [14, 15]. In comparison with chromatographic methods, spectrophotometric methods utilize less sophisticated instruments and consume low volumes of organic solvents making them functional alternatives [16]. To the best of authors' knowledge, there is no reported spectrophotometric method in the literature for the simultaneous determination of CHZ, CRZ, HAL, IMP, and PTN. Therefore, a simple method based on chemometrics for their simultaneous determination was developed and validated using UV-Vis data.

UV-Vis spectrophotometry is a well-established fast, green, and simple analytical technique that can be used for direct analysis with no need to prior tedious separation steps. The main challenge that might arise is the presence of highly overlapped spectra of the compounds to be analyzed as is the case in multicomponent mixtures like the five drugs in this study. In such instances, conventional spectrophotometric techniques such as ratio spectra [17, 18] cannot be used to resolve such spectra. Therefore, multivariate calibration models such as multivariate curve resolution (MCR) may be the method of choice to resolve such kind of severe spectral overlap. Such models have been reported to be a valid alternative to HPLC for pharmaceutical analysis [19, 20]. MCR is a mathematical algorithm first proposed in 1995 [21]. It has been reported to be more advantageous over other multivariate calibration techniques by being able to provide detailed information about concentration and spectral profiles of the compounds analyzed in the mixtures studied and has the ability of the quantitative analysis in the presence of the unknown interference [22].

The algorithm has been successfully used in different applications such as spectrophotometric pharmaceutical analysis [13, 23–25]. For further information about MCR, readers can refer to [26].

In this study, we developed and validated a spectrophotometric method for the simultaneous determination of the above mentioned CNS pharmaceuticals with severely overlapped spectra using MCR-ALS. The method was employed for the analysis of different commercial dosage forms without any preliminary separation step.

## 2. Experimental

### 2.1. Instrumentation and Software

UV spectra were acquired using a UV-1800 Shimadzu double-beam spectrophotometer (Shimadzu, Kyoto, Japan) using a 1.0 cm quartz cell. Absorbances were automatically acquired in the range of 200–400 nm, scanning speed of 2,800 nm/min, and bandwidth of 1 nm. Data acquisition was conducted using Shimadzu UV-Probe 2.62 software. The MCR-ALS model was developed via using MCR-ALS GUI 2.0 software with Matlab 2015a [27] freely accessible at http://www.mcrals.info; MCR-ALS calculations have been performed and obtained.

### 2.2. Chemicals and Reagents

The supplied CNS pure standards of CHZ, CRZ, HAL, IMP, and PTN were obtained from Sigma–Aldrich (Steinheim, Germany) and confirmed to contain ≥98%, for all analytes. HPLC-grade methanol purchased from Merck (Darmstadt, Germany) was also used. Ultrapure water (18.2 MΩ) was purified by the Pure Lab Ultra water system (ELGA, High Wycombe, UK) which was used for the entire sample preparation procedure.

Phentyin® capsules (El-Nile Co., Egypt), labeled to contain 50 mg of PTN per capsule; Haloperidol® ampoule (Sunny Pharmaceuticals, Egypt), each ampoule was labeled to contain 5 mg of HAL per 1 mL; Imipramine® tablets (ACDIMA, Egypt), labeled to contain 25 mg imipramine per tablet; Carbapex® tablets (Multi-Apex Pharma, Egypt) labeled to contain 200 mg CRZ per tablet; and Neurazine® tablets (Misr Company for Pharmaceuticals, Egypt), labeled to contain 100 mg CHZ per tablet, were used.

### 2.3. Standard Solutions and Calibration

Standard stock solutions were prepared individually in methanol by dissolving 10 mg of each standard in 10 mL methanol (i.e., 1000 *μ*g/mL) and stored in dark at 4°C. Working standard solutions were prepared by appropriate dilution in ultrapure water. A five-factor five-level experimental design [28] was employed to develop the calibration model in the concentration range of 0.3–5 *μ*g/mL for CHZ, 0.3–15 *μ*g/mL for CRZ, 0.5–10 *μ*g/mL for HAL, 0.5–10 *μ*g/mL for IMP, and 3–20 *μ*g/mL for PTN. A set of 25 calibration mixtures were prepared. The validation set was developed using the same experimental design used to build the calibration mixtures. A validation set of further 15 samples containing the five analytes with different concentrations within the calibration range were equivalently prepared. Calibration and validation set concentration design are represented in Table 1.

The UV spectra of all samples were scanned over the wavelength range of 200–400 nm with data points collected every 1 nm, and the data were exported into Matlab for the following handling for the MCR-ALS model. Five components were used for MCR-ALS determination of all analytes.

### 2.4. Analysis of the Commercial Pharmaceutical Formulations

Ten tablets or the content of ten hard gelatin capsules of each commercial dosage form were separately mixed and weighed. A weight portion of each product equivalent to 50 mg of CHZ, CRZ, IMP, and PTN was dissolved individually in 35 mL methanol using ultrasonication for 30 min, then the solution was left to cool down, and the volume was completed to 50 mL with methanol. All solutions were filtered through 0.45 *μ*m membrane filters. Appropriate dilutions were carried out in ultrapure water to prepare the working solutions.

For Haloperidol ampules, ten ampules were mixed together in 50 mL volumetric flask and the solution was completed to volume with methanol. The solution was then filtered through 0.45 *μ*m membrane filters, and further dilutions were made in ultrapure water to obtain working solutions.

### 2.5. Multivariate Calibration Analysis (MCR-ALS)

A brief description of MCR-ALS will be provided. For more details about the technique, readers are referred to [29]. MCR obtains significant information about pure compounds in a mixture via mathematical bilinear model decomposition of the data matrix according to the following equation:

(1) $$\begin{array}{c} D=CS^{T}+E, \end{array}$$

where *D* is the experimental data matrix containing all the spectra of all components of the mixture, *C* is the pure concentration profiles of each compound in the mixture, *S*^*T*^ is the matrix of the corresponding pure spectra, and *E* is the residuals matrix (i.e., data that were not expressed by the model or error matrix) [29].

The first step in MCR-ALS is to estimate the number of components, which can be simply obtained using singular value decomposition. An iterative ALS procedure is used to achieve resolution. This procedure is initialized using an initial estimation of the spectral or concentration profiles for each analyte. These initial estimates can be obtained using different algorithms such as evolving factor analysis (EFA) [30] or simple to use interactive self-modeling mixture analysis (SIMPLISMA) [31]. In this work, the known pure spectra of each individual analyte were used for initial estimation.

Several constraints can be applied for the optimization of the ALS such as correlation, closure, nonnegativity, and unimodality constraints [32]. In this work and during the ALS optimization, the nonnegativity constraint was applied to spectral and concentration profiles.

The nonnegativity constraint forces the concentration and/or spectral profiles to be ≥zero. In addition, correlation constraint was applied during the optimization process. Correlation constraint helps to build the MCR-ALS calibration model that enables the prediction of all mixture compounds even if unknown interfering compounds are there [33].

Once the abovementioned steps are completed, the developed calibration model is then used to predict the concentration in the validation and test set samples. ALS iteration will be repeated after updating the prediction results obtained till a certain convergence criterion is achieved. Usually, convergence is achieved when the difference of the root-mean-square error of residual matrix *E* of two consecutive cycles is lower than a previously set threshold value (usually >0.1%). The percentage of lack of fit equation (2) can be used to evaluate the developed MCR-ALS model:

(2) $$\begin{array}{c} \text{lack of fit}\left(\%\right)=100\sqrt{\frac{\sum_{i,j}e_{i}^{2},j}{\sum_{i,j}d_{i}^{2},j}}, \end{array}$$

where *e*_*ij*_ is the difference between experimental data input and data predicted by the model and *d*_*ij*_ is an element of the data matrix *D*.

### 2.6. Validation of the Model

To evaluate the quality of prediction of the developed MCR-ALS model, a group of external validation samples were used. Several figures of merit were calculated according to the following equations to describe the quality of validation results.

Root-mean-square error of prediction (RMSEP):

(3) $$\begin{array}{c} \text{RMSEP}=\sqrt{\frac{\sum_{i=1}^{n}{\left(c_{i}-{\hat{c}}_{i}\right)}^{2}}{n}}. \end{array}$$

Bias:

(4) $$\begin{array}{c} \text{bias}=\frac{\sum_{i=1}^{n}\left(c_{i}-{\hat{c}}_{i}\right)}{n}. \end{array}$$

The standard error of prediction (SEP):

(5) $$\begin{array}{c} \text{SEP}=\sqrt{\frac{\sum_{i=1}^{n}{\left(c_{i}-{\hat{c}}_{i}-\text{bias}\right)}^{2}}{n-1}}. \end{array}$$

Relative percentage error in the concentration predictions RE (%):

(6) $$\begin{array}{c} \text{RE}\left(\%\right)=100\sqrt{\frac{\sum_{i=1}^{n}{\left(c_{i}-{\hat{c}}_{i}\right)}^{2}}{\sum_{i=1}^{n}c_{i}^{2}}}, \end{array}$$

where *c*_*i*_ and *ĉ*_*i*_ are known and predicted analyte concentrations in sample *i*, respectively, and *n* is the number of validation samples.

Moreover, the slope, intercept, and correlation coefficient were calculated for a linear regression fit performed between the known and predicted concentrations for each compound in the mixture.

## 3. Results and Discussion

### 3.1. Spectral Characteristics and Selection of the Wavelength Range

**Figure 1:**
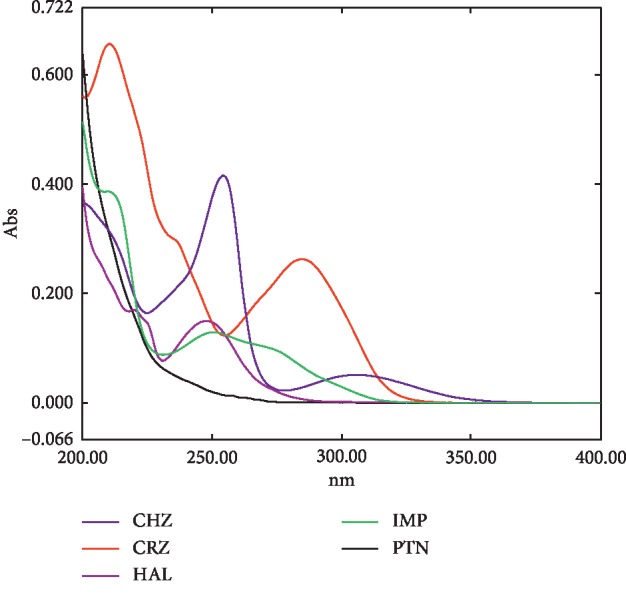
UV absorption spectra of 5 *μ*g/mL of chlorpromazine (CHZ), carbamazepine (CRZ), haloperidol (HAL), imipramine (IMP), and phenytoin (PTN).

Figure 1 demonstrates the pure UV absorption spectra of the five analytes CHZ, CRZ, HAL, IMP, and PTN at the concentration of 5 *μ*g/mL of each analyte. As shown below, the spectra are extremely overlapped along the entire range of absorption. Therefore, the use of univariate or conventional spectrophotometric methods is not feasible for the quantitative analysis of such mixture. Thus, the proposed MCR-ALS method was utilized to resolve this complex mixture.

The quality of multivariate analysis is highly dependent on the selection of the optimum wavelength range [34]. Absorbance spectra in the range of 200–220 nm were excluded as they contained noise. In addition, the range of 270–320 nm was also excluded as HAL and PTN did not show significant absorption bands in this region. Accordingly, the wavelength range of 220–270 nm was selected employed for developing the model.

### 3.2. MCR-ALS Model

A multilevel multifactor experimental design [28] was employed to build the calibration model. For every individual analyte, five concentration levels were used. The chosen design provided factors that are orthogonal to each other and spanned each other's calibration space symmetrically.

The initial estimation of the pure spectral profiles of the target analytes employed singular value decomposition and revealed five major components in the data matrix. In order to test the MCR-ALS resolution and to decrease the rotational ambiguities effects, the pure spectra of the target analytes were used as initial estimates to check the MCR-ALS resolution and reduce the model rotational ambiguity effects [29]. SIMPLISMA was used to calculate the initial spectral profiles estimates. The MCR-ALS model was applied to the data matrix using nonnegativity constraint in both spectral and concentration profiles and a fast nonnegativity constrained least squares algorithm (*fnnls*) [35] was employed. Moreover, a correlation constraint was also used, and the variable containing the quantitative information of the five target analytes was selected. Satisfactory results were obtained with a low lack of fit (% lof) of 0.3541. The convergence criterion was set at 0.1% and the maximum number of iterations was 50; however, only 7 iterations were required to achieve convergence in all tested mixtures.

**Figure 2:**
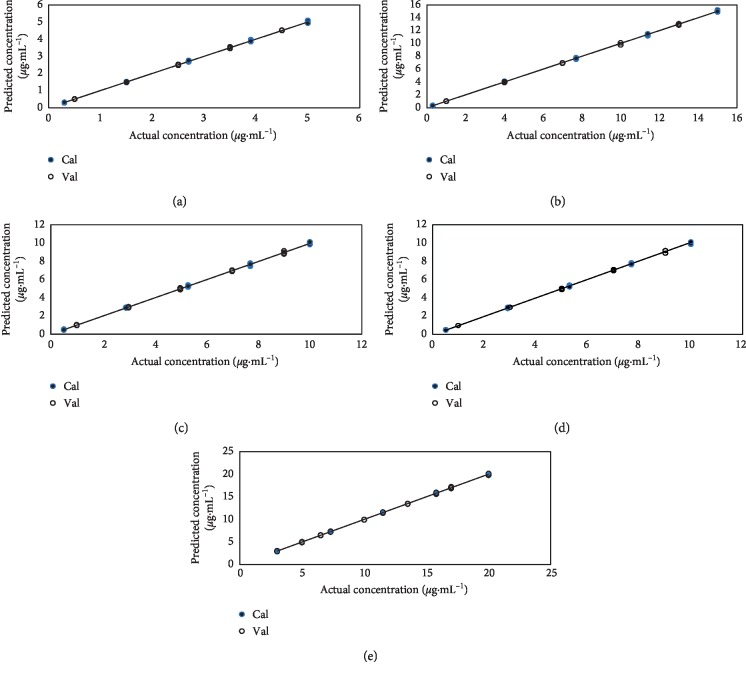
Scatter plot of actual analytes concentration versus the MCR-ALS-predicted concentrations of (a) CHZ, (b) CRZ, (c) HAL, (d) IMP, and (e) PTN.

The scatter plot of the predicted MCR-ALS concentrations versus the actual concentrations is shown in Figure 2, with correlation coefficients (*r*^2^ > 0.9993) for all analytes, indicating the good perdition ability of the developed model.  Table 2 shows the figures of merit of the regression model of the calibration set. The results show excellent prediction power with correlation coefficients (*r*^2^ > 0.9993) and low relative error RE (%) = 1.14, 1.00, 1.65, 0.92 and 0.61% for HAL, IMP, CHZ, CRZ, and PTN, respectively).

### 3.3. Method Validation

The quantitative prediction capability of the established model was tested by applying the model for the prediction of the concentration of CHZ, CRZ, HAL, IMP, and PTN in an external validation set of 15 synthetic mixtures with different concentrations within the calibration range of each analyte (Table 1). This was done by means of using the same identical constraints applied for the calibration set. Various parameters (RMSEP, SEP, RE (%), and *r*^2^) were calculated to judge the predictive behavior of the proposed model. The validation results are presented in Table 3.

#### 3.3.1. Linearity

The absorption spectra of each drug and their mixtures as well were checked for their linearity. The results are linear in the concentration range of 0.3–5 *μ*g/mL for CHZ, 0.3–15 *μ*g/mL for CRZ, 0.5–10 *μ*g/mL for HAL, 0.5–10 *μ*g/mL for IMP, and 3–20 *μ*g/mL for PTN. The model showed excellent prediction for the validation set represented in the good correlation coefficients ranging between 0.9993 and 0.9998 for all analytes. Figure 2 shows the regression plots of the MCR-ALS-predicted analyte concentrations versus the actual concentrations. In addition, low relative errors (RE (%)) between 0.67 and 1.42% were obtained expressing the quality of fit of the entire calibration data.

#### 3.3.2. Accuracy

The accuracy of the developed method was evaluated using the standard addition method. The percent recoveries results were satisfactory ranging from 99.3% to 100.1% with %SDs not higher than 1.6% (Table 3). These results confirmed that the excipients in commercial formulations do not interfere with the determination of the studied analytes.

#### 3.3.3. Precision

The intraday precision and interday precision of the proposed method were assessed by analyzing three concentration levels: low, intermediate, and high (as indicated in Table 3) of the studied drugs within the same day for intraday precision and at three consecutive days for interday precision. The lower values of %RSD (˂1.6) indicated good precision of the developed method (Table 3).

#### 3.3.4. Limits of Detection (LOD) and Limits of Quantifications

Limits of detection (LODs) and limits of quantifications (LOQs) were calculated following the methodology described in [36]. In this work, LODs were in the range of 0.06 to 0.14 *μ*g·mL^−1^, while the LOQs ranged from 0.19 to 0.42 *μ*g·mL^−1^. Table 3 shows the values obtained.

The developed model demonstrated satisfactory validation results.

### 3.4. Literature Comparison

This study established a spectrophotometric method using MCR-ALS for simultaneous determination of imipramine, carbamazepine, chlorpromazine, haloperidol, and phenytoin in commercial formulations. An overview of analytical methods reported for the determination of CNS affecting drugs in pharmaceutical dosage forms revealed that all reported methods [6–11] either use or generate harmful solvents. Moreover, LODs of the proposed method were similar to or even better than those of the reported methods. Overall, the comparison of the results showed that the presented method is eco-friendly and more sensitive than the reported methods. Furthermore, the developed method is economic due to saving in solvent consumption and minimizing in preparation time and thus can be applied for the routine analysis of the studied pharmaceuticals without harming the environment.

### 3.5. Analysis of Pharmaceutical Products

The developed model was applied for the analysis of the studied pharmaceuticals in different commercial pharmaceutical dosage forms including tablets, capsules, and ampules. Six replicate determinations were performed. Satisfactory results were obtained (Table 4) which were in good agreement with the label claims.

Finally, the obtained MCR-ALS results were statistically compared with a reported HPLC method [11] for the simultaneous determination of the five target analytes using Student's *t* test and *F* ratio at 95% confidence level. The results showed no significant difference regarding accuracy and precision (Table 4).

## 4. Conclusion

This work presents a fast, simple, eco-friendly, precise, and accurate method for the simultaneous spectrophotometric analysis of five CNS pharmaceuticals in different dosage forms such as tablets, capsules, and ampoules. The developed MCR-ALS model results were compared with a reported HPLC method, and there was no significant difference between the proposed and the reference method regarding the accuracy and precision. The proposed chemometric method has demonstrated its efficiency to be a valid eco-friendly alternative to the chromatographic techniques for the determination of pharmaceuticals in different dosage forms. Therefore, it can be used for quality control testing without the need for sample preparation and costly solvents.

## Figures and Tables

**Table 1:** The concentration matrix used for the preparation of the calibration and validation sets of CHZ, CRZ, HAL, IMP, and PTN.

Sample no.	Calibration set (*μ*g/mL)					Validation set (*μ*g/mL)				
	HAL	IMP	CHZ	CRZ	PTN	HAL	IMP	CHZ	CRZ	PTN
1	5.3	5.3	2.7	7.7	11.5	9.0	7.0	0.5	13.0	17.0
2	5.3	0.5	0.3	15.0	7.3	7.0	5.0	4.5	1.0	5.0
3	0.5	0.5	5.0	4.0	20.0	5.0	9.0	4.5	4.0	13.5
4	0.5	10.0	1.5	15.0	11.5	9.0	9.0	0.5	10.0	5.0
5	10	2.9	5.0	7.7	7.3	9.0	1.0	3.5	4.0	10.0
6	2.9	10.0	2.7	4.0	7.3	1.0	7.0	0.5	7.0	13.5
7	10.0	5.3	1.5	4.0	15.8	7.0	1.0	2.5	10.0	13.5
8	5.3	2.9	1.5	11.4	20.0	1.0	5.0	3.5	1.0	6.5
9	2.9	2.9	3.9	15.0	15.8	5.0	7.0	3.5	4.0	5.0
10	2.9	7.7	5.0	11.4	11.5	7.0	7.0	1.5	1.0	6.5
11	7.7	10.0	3.9	7.7	20.0	7.0	3.0	0.5	4.0	10.0
12	10.0	7.7	2.7	15.0	20.0	3.0	1.0	1.5	7.0	5.0
13	7.7	5.3	5.0	15.0	3.0	1.0	3.0	2.5	13.0	5.0
14	5.3	10.0	5.0	0.3	15.8	3.0	5.0	0.5	4.0	17.0
15	10.0	10.0	0.3	11.4	3.0	5.0	1.0	2.5	13.0	6.5
16	10.0	0.5	3.9	0.3	11.5					
17	0.5	7.7	0.3	7.7	15.8					
18	7.7	0.5	2.7	11.4	15.8					
19	0.5	5.3	3.9	11.4	7.3					
20	5.3	7.7	3.9	4.0	3.0					
21	7.7	7.7	1.5	0.3	7.3					
22	7.7	2.9	0.3	4.0	11.5					
23	2.9	0.5	1.5	7.7	3.0					
24	0.5	2.9	2.7	0.3	3.0					
25	2.9	5.3	0.3	0.3	20.0					

**Table 2:** Figures of merit of the MCR-ALS regression model for the calibration set of HAL, IMP, CHZ, CRZ, and PTN.

Parameters	HAL	IMP	CHZ	CRZ	PTN
Calibration range (*μ*g·mL^−1^)	0.5–10	0.5–10	0.3–5	0.3–15	3–20
Intercept (a)	−3.11 × 10^−14^	−5.15 × 10^−14^	8.88 × 10^−15^	3.64 × 10^−14^	2.49 × 10^−14^
Standard error of intercept	2.9 × 10^−2^	2.9 × 10^−2^	1.7 × 10^−2^	3.3 × 10^−2^	3.7 × 10^−2^
Slope (b)	1.0000	1.0000	1.0000	1.0000	1.0000
Standard error of slope	4.66 10^−3^	4.79 × 10^−3^	5.48 × 10^−3^	3.53 × 10^−3^	2.85 × 10^−3^
RMSECV	3.89 × 10^−2^	3.32 × 10^−2^	2.79 × 10^−2^	5.03 × 10^−2^	4.70 × 10^−2^
SEP	3.81 × 10^−2^	3.25 × 10^−2^	2.74 × 10^−2^	4.93 × 10^−2^	4.60 × 10^−2^
Bias	7.21 × 10^−3^	2.32 × 10^−3^	−4.81 × 10^−3^	5.68 × 10^−3^	−1.44 × 10^−3^
RE (%)	1.14	1.004	1.65	0.92	0.61
LOD (*μ*g·mL^−1^)	0.13	0.14	0.09	0.06	0.72
LOQ (*μ*g·mL^−1^)	0.38	0.42	0.28	0.19	2.18
Correlation coefficient (*r*^2^)	0.9995	0.9995	0.9993	0.9997	0.9998

**Table 3:** Figures of merit of the MCR-ALS regression model for the validation set of CHZ, CRZ, HAL, IMP, and PTN.

Parameters	HAL	IMP	CHZ	CRZ	PNT
Accuracy (mean ± SD)^a^	99.3 ± 1.45	99.8 ± 1.55	99.8 ± 1.13	100.1 ± 1.45	99.9 ± 0.65
Precision repeatability (RSD%)^b^	1.09	1.32	0.89	0.67	0.78
Intermediate precision (RSD%)^c^	1.46	1.56	1.13	1.44	0.65
RMSEP	6.56 × 10^−2^	6.17 × 10^−2^	1.65 × 10^−2^	7.81 × 10^−2^	5.54 × 10^−2^
SEP	6.34 × 10^−2^	5.96 × 10^−2^	1.59 × 10^−2^	7.54 × 10^−2^	5.35 × 10^−2^
Bias	6.96 × 10^−3^	−1.74 × 10^−2^	−2.36 × 10^−3^	2.07 × 10^−2^	6.74 × 10^−3^
RE (%)	1.33	1.35	0.75	1.42	0.67
Correlation coefficient (*r*^2^)	0.9994	0.9995	0.9997	0.9996	0.9998

^a^The mean and standard deviation for 15 determinations. ^b^The intraday relative standard deviation (*n* = 3), an average of three different concentration repeated three times within the same day. ^c^The interday relative standard deviation (*n* = 3), an average of three different concentration repeated three times in three different days. Low concentrations: 1 *μ*g/mL for HAL, IMP, and CRZ, 0.5 *μ*g/mL for CHZ, and 5 *μ*g/mL for PTN. Intermediate concentrations: 5 *μ*g/mL for HAL, IMP, and CRZ, 2.5 *μ*g/mL for CHZ, and 10 *μ*g/mL for PTN. High concentration: 10 *μ*g/mL for HAL, IMP, and CRZ, 5 *μ*g/mL for CHZ, and 20 *μ*g/mL for PTN.

**Table 4:** Determination of the studied drugs in commercial products by the MCR-ALS method, the proposed method, and the reported HPLC method.

	MCR-ALS	HPLC
Analytes		
HAL (Haloperidol ampoule)		
Mean + SD	98.8 ± 1.38	99.8 ± 1.31
*t*	1.19	—
*F*	1.12	—
IMP (Imipramine tablets)		
Mean + SD	98.2 ± 0.82	99.8 ± 0.71
*t*	1.29	—
*F*	1.33	—
CHZ (Neurazibe tablets)		
Mean + SD	99.06 ± 0.96	99.9 ± 0.83
*t*	1.61	—
*F*	1.35	—
Compound		
CRZ (Carbapex tablets)		
Mean + SD	99.6 ± 0.39	99.8 ± 0.49
*T*	0.51	—
*F*	1.56	—
PTN (Phenytin capsules)		
Mean + SD	99.5 ± 0.13	99.7 ± 0.14
*T*	1.94	—
*F*	1.13	—

The reference HPLC published method used the C8 (250 × 4.6 mm, 5.0 *μ*m) column at 30°C, and the mobile phase was composed of acetonitrile and sodium dihydrogenophosphate buffer used in gradient elution mode at 1.5 mL·min^−1^ flow rate. SD: standard deviation of the mean of the percentage recovery from the label claim amount for 6 determinations. Theoretical values for *t* and *F* at (*p*=0.05) are 2.23 and 5.05, respectively.

## Data Availability

The data used to support the findings of this study are available from the corresponding author upon request.
